# E-waste hazard: The impending challenge

**DOI:** 10.4103/0019-5278.43263

**Published:** 2008-08

**Authors:** Violet N. Pinto

**Affiliations:** Department of Community Medicine, Dr. D.Y. Patil Medical College and Hospital, Nerul, Navi Mumbai-400 706, India

**Keywords:** E-waste, environmental hazard, occupational hazard

## Abstract

Electronic waste or e-waste is one of the rapidly growing problems of the world. E-waste comprises of a multitude of components, some containing toxic substances that can have an adverse impact on human health and the environment if not handled properly. In India, e-waste management assumes greater significance not only due to the generation of its own e-waste but also because of the dumping of e-waste from developed countries. This is coupled with India's lack of appropriate infrastructure and procedures for its disposal and recycling. This review article provides a concise overview of India's current e-waste scenario, namely magnitude of the problem, environmental and health hazards, current disposal and recycling operations, existing legal framework, organizations working on this issue and recommendations for action.

## INTRODUCTION

The production of electrical and electronic equipment (EEE) is one of the fastest growing global manufacturing activities. Rapid economic growth, coupled with urbanization and a growing demand for consumer goods, has increased both the consumption and the production of EEE.[1] The Indian information technology (IT) industry has been one of the major drivers of change in the economy in the last decade and has contributed significantly to the digital revolution being experienced by the world. New electronic gadgets and appliances have infiltrated every aspect of our daily lives, providing our society with more comfort, health and security and with easy information acquisition and exchange.[2] The knowledge society however is creating its own toxic footprints.

The same hypertechnology that is hailed as a ‘crucial vector’ for future modern societal development has a not-so-modern downside to it: electronic waste (e-waste).[3]

E-waste broadly covers waste from all electronic and electrical appliances and comprises of items such as computers, mobile phones, digital music recorders/players, refrigerators, washing machines, televisions (TVs) and many other household consumer items.[2]

The increasing ‘market penetration’ in the developing countries, ‘replacement market’ in the developed countries and ‘high obsolescence rate’ make e-waste one of the fastest waste streams.[4] This new kind of waste is posing a serious challenge in disposal and recycling to both developed and developing countries. While having some of the world's most advanced high-tech software and hardware developing facilities, India's recycling sector can be called medieval.[3] The dumping of e-waste, particularly computer waste, into India from developed countries[5] (‘green passport’ according to Gutierrez[6]), because the latter find it convenient and economical to export waste, has further complicated the problems with waste management.

All this has made e-waste management an issue of environment and health concern.

## MAGNITUDE OF THE PROBLEM

Studies so far reveal that the total e-waste generation in India is approximately 1,46,000 tonnes[7–9] to 3.3 lakh tonnes a year and is expected to touch 4.7 lakh tonnes by 2011.[10] ‘The projected growth for e-waste generation for India is about 34% year on year’ says Sinha (Associate Director of Toxics Link).[11]

Of the total e-waste generated in the country, western India accounts for the largest population at 35%, while the southern, northern and eastern regions account for 30, 21 and 14%, respectively. The top states in order of highest contribution to waste electrical and electronic equipment (WEEE) include Maharashtra, Andhra Pradesh, Tamil Nadu, Uttar Pradesh, West Bengal, Delhi, Karnataka, Gujarat, Madhya Pradesh and Punjab. The city-wise ranking of the largest WEEE generators is Mumbai, Delhi, Bangalore, Chennai, Kolkatta, Ahmedabad, Hyderabad, Pune, Surat and Nagpur.[8]

Total WEEE generation in Maharashtra is 20,270.6 tonnes, of which Navi Mumbai contributes 646.48 tonnes, Greater Mumbai 11,017.06 tonnes, Pune 2584.21 tonnes and Pimpri-Chinchwad 1032.37 tonnes. An estimated 30,000 computers become obsolete every year from the IT industry in Bangalore alone.[8] Home to more than 1200 foreign and domestic technology firms, Bangalore figures prominently in the danger list of cities faced with e-waste hazard. As many as 1000 tonnes of plastics, 300 tonnes of lead, 0.23 tonnes of mercury, 43 tonnes of nickel and 350 tonnes of copper are annually generated in Bangalore.[9] While on the basis of scrap handled by the Delhi-based scrap dealers, their total number of personal computers (PCs) meant for dismantling would be around 15,000 per year. This figure does not include PCs handled by large dealers who get scraps from foreign sources.[12] Mumbai, the financial nerve-center of India, alone throws away 19,000 tonnes of electronic waste a month, excluding the large e-waste it imports from developed nations through its port.[11]

Besides the domestic e-waste generated, an additional 50,000 MT a year is illegally imported into the country.[10] In a single month, there is a reported case of import of 30 MT of e-waste at the Ahmedabad port.[12]

While northern India is not a leading generator, it happens to be the leading processing center of e-waste in the country. There are only two formal recyclers in the south of India (at Chennai and Bangalore) and one in western India. Currently, there are no formal recyclers operating in the north or the east.[10,13] Over 1 million poor people in India are involved in the manual recycling operations.[12] Most of the people working in this recycling sector are the urban poor with very low literacy levels and hence very little awareness regarding the hazards of e-waste toxins. There are a sizeable number of women and children who are engaged in these activities and they are more vulnerable to the hazards of this waste.[2] A comprehensive study is yet to be made of the health problems of women and children employed by the scrap dealers.[14]

The main sources of computer usage and thereby e-waste generations are the business sector (government departments, public or private sector, multinational corporation offices, etc.), accounting for 78% of the total installed PCs today. Other sources are individual households (22%), foreign embassies, PC manufacturing units, PC retailers, secondary markets of old PCs and imported electronic scrap of other countries.[12]

The following three categories of WEEE account for almost 90% of the generation:[8]

1. Large household appliances: 42%,
2. Information and communications technology equipment: 33.9% and
3. Consumer electronics: 13.7%.

## WHAT IS E-WASTE?

Electronic waste or e-waste is the term used to describe old, end-of-life electronic appliances such as computers, laptops, TVs, DVD players, mobile phones, mp3 players, etc., which have been disposed by their original users.[8]

E-waste has been categorized into three main categories, i.e., Large Household Appliances, IT and Telecom and Consumer Equipment. Refrigerator and washing machine represent large household appliances; PC, monitor and laptop represent IT and Telecom, while TV represents Consumer Equipment.

Each of these e-waste items has been classified with respect to 26 common components found in them. These components form the ‘building blocks’ of each item and therefore they are readily ‘identifiable’ and ‘removable.’ These components are metal, motor/ compressor, cooling, plastic, insulation, glass, LCD, rubber, wiring/electrical, concrete, transformer, magnetron, textile, circuit board, fluorescent lamp, incandescent lamp, heating element, thermostat, brominated flamed retardant (BFR)-containing plastic, batteries, CFC/HCFC/HFC/HC, external electric cables, refractory ceramic fibers, radioactive substances and electrolyte capacitors (over L/D 25 mm).

The composition of WEEE/e-waste is very diverse and differs in products across different categories. It contains more than 1000 different substances, which fall under ‘hazardous’ and ‘non-hazardous’ categories. Broadly, it consists of ferrous and non-ferrous metals, plastics, glass, wood and plywood, printed circuit boards, concrete and ceramics, rubber and other items. Iron and steel constitutes about 50% of the WEEE followed by plastics (21%), non-ferrous metals (13%) and other constituents. Non-ferrous metals consist of metals like copper, aluminium and precious metals, e.g. silver, gold, platinum, palladium, etc. The presence of elements like lead, mercury, arsenic, cadmium, selenium and hexavalent chromium and flame retardants beyond threshold quantities in WEEE/e-waste classifies them as hazardous waste.[4]

The electronic and electrical goods are largely classified under three major heads, as: ‘white goods,’ comprising of household appliances like air conditioners, dishwashers, refrigerators and washing machines; ‘brown goods,’ comprising of TVs, camcorders, cameras, etc.; ‘grey goods,’ like computers, printers, fax machines, scanners, etc. The grey goods are comparatively more complex to recycle due to their toxic composition.[2]

## HEALTH AND ENVIRONMENTAL IMPACT OF E-WASTE

EEEs are made of a multitude of components, some containing toxic substances that have an adverse impact on human health and the environment if not handled properly. Often, these hazards arise due to the improper recycling and disposal processes used.[8] It can have serious repercussions for those in proximity to places where e-waste is recycled or burnt. Waste from the white and brown goods is less toxic as compared with grey goods. A computer contains highly toxic chemicals like lead, cadmium, mercury, beryllium, BFR, polyvinyl chloride and phosphor compounds.[2]

Table 1: Environment and health hazards.

**Table 1:** Environment and health hazards.[12,13]

Computer/e-waste component	Process	Potential occupational hazard	Potential environmental hazard
Cathode ray tubes	Breaking, removal of copper yoke and dumping	Silicosis Cuts from CRT glass Inhalation or contact with phosphor containing cadmium or other metals	Lead, barium and other heavy metals leaching into ground water and release of toxic phosphor
Printer circuit boards	Desoldering and removing computer chips	Tin and lead inhalation Possible brominated dioxin, beryllium, cadmium and mercury inhalation	Air emission of the same substances
Dismantled printed circuit board processing	Open burning of waste boards	Toxicity of workers and nearby residents rom tin, lead, brominated dioxin, beryllium, cadmium and mercury inhalation	Tin and lead contamination of immediate environment, including surface and ground waters, brominated dioxins, beryllium, cadmium and mercury inhalation
Chips and other gold-plated compounds	Chemical stripping using nitric and hydrochloric acid along riverbanks	Acid contact with eyes, skin may result in permanent injury Inhalation if mists and fumes of acids, chlorine and sulfur dioxide gases can cause respiratory irritation to severe effects, including pulmonary edema, circulatory failure and death	Hydrocarbons, heavy metals, brominated substances etc. discharged directly into river and banks. Acidifies the river destroying fish and flora
Plastics from the computer and peripherals	Shredding and low-temperature melting	Probable hydrocarbon, brominated dioxin and PAH exposure to workers living in the burning works area	Emission of brominated dioxins and heavy metals and hydrocarbons
Secondary steel or copper and precious metal smelting	Furnace recovers steel or copper from waste	Exposure to dioxins and heavy metals	Emission of dioxins and heavy metals
Wires	Open burning to recover copper	Brominated and chlorinated dioxin and PAH exposure to workers living in the burning works area	Hydrocarbon and ashes, including PAHs discharged into air, water and soil

### Lead

exerts toxic effects on various systems in the body such as the central (organic affective syndrome) and peripheral nervous systems (motor neuropathy), the hemopoietic system (anemia), the genitourinary system (capable of causing damage to all parts of nephron) and the reproductive systems (male and female).[15]

### Mercury

causes damage to the genitourinary system (tubular dysfunction), the central and peripheral nervous systems as well as the fetus. When inorganic mercury spreads out in the water, it is transformed into methylated mercury, which bio-accumulates in living organisms and concentrates through the food chain, particularly by fish.[4,15,16]

### Cadmium

is a potentially long-term cumulative poison. Toxic cadmium compounds accumulate in the human body, especially in the kidneys. There is evidence of the role of cadmium and beryllium in carcinogenicity.[17–19]

### Polycyclic aromatic hydrocarbons (PAH)

Affects lung, skin and bladder. Epidemiological studies in the past on occupational exposure to PAH provide sufficient evidence of the role of PAH in the induction of skin and lung cancers.[17,19]

## EXISTING LEGISLATIONS AND POLICY RELATED TO E-WASTE[20]

Draft Hazardous Materials (Management, Handling and Transboundary movement) Rules, 2007 (dated: September 28, 2007), part of the Environment Protection Act, 1986.

India is a signatory to the Basal Convention. (Basel Convention is the United Nations Environment Programme) on the control of Transboundary Movement of Hazardous wastes and their disposal.

There is no policy on e-waste, although some parts of computers could be considered as hazardous waste.

## ORGANIZATIONS/NETWORKS WORKING ON E-WASTE ISSUES

### Within India

#### 1. Knowledge bank for e-waste management in India.[21]

The Asia Pro Ecoprogramme supported by the European Commission is dedicated to the environmental performance in Asian Economic sectors through the exchange of environmental policies, technologies and practices and to promote sustainable investment and trade between the European Union Member States and South Asia, South-East Asia and China.

#### 2. The E-waste Guide, India (www.ewaste.in).

An Initiative of the Indo–German–Swiss Partnership [Ministry of Environment and Forests, German Federal Ministry for Economic Cooperation and Development and Swiss State Secretariat for Economic Affairs] It is designed to serve as an information resource on e-waste as well as a common collaborative work platform for stakeholders.

#### 3. National Solid Waste Association of India (NSWAI) (www.nswai.com).

A leading professional non-profit organization in the field of solid-waste management, including toxic and hazardous waste and also biomedical waste in India. It was formed in 1996. Its objectives include development of solid-waste management as a profession, research and development, development of expertise, standards and goods practices with regards to solid-waste management. Some of the others include improvement in legislation and creating awareness and community involvement.

#### 4. Toxics Link (www.toxicslink.org).

A Delhi-based environment activist group with a mission of working for environmental justice and freedom from toxics. It is also actively involved in creating public awareness on environmental issues through publications, reports, articles and environment news bulletins besides organizing various events.

5. Others are stEP Workweb, WEEE Forum, Clean India, Indian Environmental Society, INDIA HABITAT CENTRE and Microbial Biotechnology Area of Tata Energy Research Institute.

### International networks

#### 1. Silicon Valley Toxics Coalition

Formed in 1982, located in San Jose, California, it is a diverse grassroots coalition that engages in research and advocacy and is organized around the environmental and human health problems caused by the rapid growth of the high-tech electronics industry. The Coalition has built a united campaign of allies, including community residents, consumers, electronics and technology workers and government policy makers to raise the environmental consciousness and performance of the high-tech sector.

#### 2. The Basel Action Network (BAN)

A global network of toxics and development activist organizations that share a vision of international environmental justice. The network seeks to prevent all forms of ‘toxic trade’ – in toxic wastes, toxic products and toxic technologies. It works to prevent the globalization of the toxic chemical crisis. BAN is administered by the Secretariat services of the Asia-Pacific Environmental Exchange (APEX) based in Seattle, Washington, USA. APEX is an activity of the Tides Centre.

3. Others are the International Solid Waste Association, Solid Waste Association of North America, Environmental Protection Agency, etc.

## RECOMMENDATIONS FOR ACTION

**1. Technical interventions**[2,7,13,22,23]

- Product design and engineering interventions

The solution for the e-waste crisis lies in ‘prevention at the manufacturing source’ or the ‘precautionary principle.’ This can be done by employing waste minimization techniques and by a sustainable product design.

Waste minimization in industries involves adopting:

- Inventory management
- Production process modification
- Volume reduction
- Recovery and reuse
- Sustainable product design involves:
- Rethinking on procedures of designing the product (flat computers)
- Use of renewable material and energy
- Creating electronic components and peripherals of biodegradable material
- Looking at a green packaging option
- Utilizing a minimum packaging material

Extended Producer Responsibility is considered one of the most appropriate frameworks that amalgamates all the enlisted principles on environmental justice. This shifts the responsibility of safe disposal onto the producers. It promotes sound environmental technology and also aims at better raw material, cleaner production technology and designing for longevity.

- Restructuring recycling:

Some recycling procedures require improvements, up-gradation (both in skills and technologies) and some have to be abandoned altogether due to severe risks for health and the environment.

2. Policy-level interventions

- Clear definition of e-waste for regulation.
- Import and export regulatory regime.
- An integrated IT waste management policy

Lack of clarity on the issue of e-waste and the inability of current hazardous waste rules to govern and effectively monitor the e-waste recycling are some of the prime reasons for experts and members of civil society demanding a separate set of rules to guide and control these processes.[24]

- Take back policies

Producers must be responsible for the entire lifecycle of their products. In developed countries, several efforts have been made on this front. Several dozen cities in the states of California and Massachusettes, including San Francisco, also have passed resolutions supporting ‘producer take back’ rules. Wipro Infotech has launched an e-waste disposal service for end customers. Others offering recycling options include Dell (dell.com), HP (hp.com) and Apple (apple.com).[25]

3. Implementation and capacity building

- Legislation for collection, recycling and disposal.
- Institutional capacity building.
- Formalizing the informal recycling sector.

3.1 Technical advantage of processes improvement (restructuring recycling)

At Ash Recyclers, one of just two authorized recycling plants in Bangalore, hazardous metals are safely extracted at a special plant and everything else – down to the keys – is recycled.[9]

3.2 Protective protocol for workers in e-waste disposal

Workers are given formally recognized jobs where they can use skills and where occupational health safety (information about their occupation-related health hazards involved and self protection, protective gear and equipment and periodic medical checkups) is assured.

- Bilateral and multilateral cooperation

4. Awareness building

The current awareness regarding the existence and dangers of e-waste are extremely low, partly because the e-waste being generated is not as large as in developed countries. Urgent measures are required to address this issue.

The role of citizens in e-waste management include:

- Donating electronics for reuse, which extends the lives of valuable products and keeps them out of the waste management system for a long time.
- While buying electronic products, opting for those that are made with fewer toxic constituents, use recycled content, are energy efficient, are designed for easy upgrading or disassembly, use minimal packaging and offer leasing or take back options.
- Building of consumer awareness through public awareness campaigns is a crucial point that can attribute to a new responsible kind of consumerism.

## CONCLUSION

India is placed in a very interesting position. The need of the hour is an urgent approach to the e-waste hazard by technical and policy-level interventions, implementation and capacity building and increase in public awareness such that it can convert this challenge into an opportunity to show the world that India is ready to deal with future problems and can set global credible standards concerning environmental and occupational health.
